# Detection of Active Caspase-3 in Mouse Models of Stroke and Alzheimer's Disease with a Novel Dual Positron Emission Tomography/Fluorescent Tracer [^68^Ga]Ga-TC3-OGDOTA

**DOI:** 10.1155/2019/6403274

**Published:** 2019-01-14

**Authors:** Valeriy G. Ostapchenko, Jonatan Snir, Mojmir Suchy, Jue Fan, M. Rebecca Cobb, Blaine A. Chronik, Michael Kovacs, Vania F. Prado, Robert H. E. Hudson, Stephen H. Pasternak, Marco A. M. Prado, Robert Bartha

**Affiliations:** ^1^Robarts Research Institute, University of Western Ontario, 1151 Richmond Street, London, ON, Canada N6A 5B7; ^2^Department of Medical Biophysics, University of Western Ontario, 1151 Richmond Street, London, ON, Canada N6A 5B7; ^3^Department of Chemistry, University of Western Ontario, 1151 Richmond Street, London, ON, Canada N6A 5B7; ^4^Neuroscience Program, University of Western Ontario, 1151 Richmond Street, London, ON, Canada N6A 5B7; ^5^Department of Physics and Astronomy, University of Western Ontario, 1151 Richmond Street, London, ON, Canada N6A 5B7; ^6^Lawson Health Research Institute, 268 Grosvenor Street, London, ON, Canada N6A 4V2; ^7^Department of Physiology and Pharmacology, University of Western Ontario, 1151 Richmond Street, London, ON, Canada N6A 5B7; ^8^Department of Anatomy and Cell Biology, University of Western Ontario, 1151 Richmond Street, London, ON, Canada N6A 5B7; ^9^Department of Clinical Neurological Sciences, University of Western Ontario, 1151 Richmond Street, London, ON, Canada N6A 5B7

## Abstract

Apoptosis is a feature of stroke and Alzheimer's disease (AD), yet there is no accepted method to detect or follow apoptosis in the brain in vivo. We developed a bifunctional tracer [^68^Ga]Ga-TC3-OGDOTA containing a cell-penetrating peptide separated from fluorescent Oregon Green and ^68^Ga-bound labels by the caspase-3 recognition peptide DEVD. We hypothesized that this design would allow [^68^Ga]Ga-TC3-OGDOTA to accumulate in apoptotic cells. In vitro, Ga-TC3-OGDOTA labeled apoptotic neurons following exposure to camptothecin, oxygen-glucose deprivation, and *β*-amyloid oligomers. In vivo, PET showed accumulation of [^68^Ga]Ga-TC3-OGDOTA in the brain of mouse models of stroke or AD. Optical clearing revealed colocalization of [^68^Ga]Ga-TC3-OGDOTA and cleaved caspase-3 in brain cells. In stroke, [^68^Ga]Ga-TC3-OGDOTA accumulated in neurons in the penumbra area, whereas in AD mice [^68^Ga]Ga-TC3-OGDOTA was found in single cells in the forebrain and diffusely around amyloid plaques. In summary, this bifunctional tracer is selectively associated with apoptotic cells in vitro and in vivo in brain disease models and represents a novel tool for apoptosis detection that can be used in neurodegenerative diseases.

## 1. Introduction

Neurodegenerative diseases place a devastating burden on modern society, being among the top causes of disability and death. One of the processes leading to neurodegeneration is apoptosis, activated in many brain diseases, including Alzheimer's and Parkinson's diseases, and in the penumbra of an ischemic region. While there are established methods to measure fibrillary amyloid, tau protein, and glucose uptake by positron emission tomography (PET), and characterize brain function by magnetic resonance imaging [1–3], few methods have been developed to detect apoptosis in neurological patients [4].

Apoptosis, or programmed cell death, follows either extrinsic pathways, associated with one of the members of the tumour necrosis factor receptor family, or intrinsic pathways, associated with cytochrome C release from mitochondria [5]. Both pathways eventually merge in the terminal portion of the so-called caspase cascade. This cascade features caspase-3 activation as the final step, triggering activation of cell content proteolysis, cytoskeleton collapse [6], and DNA fragmentation [7]. Therefore, caspase-3 is considered a universal indicator of an apoptotic cell.

A number of caspase-3 activity markers have been developed in recent years, designed to either bind to or to be cleaved by active/cleaved caspase-3 (CC3) at its peptide recognition site DEVD. Highly specific CC3 inhibitors, such as ^18^F-labeled isatin derivatives, were successfully employed for PET imaging of mouse tumours [8, 9]. Synthesis of such inhibitor-based contrast agents is widely established, but their delivery to the brain has not been extensively documented. These reagents present some disadvantages, such as metabolic instability, interactions with other enzymes, and interference with apoptosis progression, since they render CC3 inactive. Other reagents engineered to be cleaved by CC3 to detect apoptosis inside the cell have used bioluminescence [10], fluorescence resonance energy transfer, [11] or reconstitution of a fluorescent protein [12]. Among the drawbacks of such approaches is the need to express an exogenous protein in the system of interest and the low tissue penetration of visible light limiting in vivo applications. Addressing this problem, compounds containing far-red or near-infrared fluorescent tags connected to their quenchers through a CC3 cleavage sequence have been designed to investigate apoptosis in vivo [13–16]. Some of these studies have also addressed the problem of delivering the contrast agent to the cell using various cell-penetrating peptides (CPP) [13, 15]. While improving imaging depth, these compounds still can be used only in cell cultures or in close-to-surface studies of tissues such as subcutaneous xenograft tumours in mice.

To improve imaging depth, a number of PET ^18^F-based tracers have also been designed to accumulate in apoptotic cells, due to either high affinity to caspase-3 [17], removal by CC3 of a short PEG-based cell-penetrating peptide [18, 19], or intracellular aggregation [20]. These have been successfully applied in dexamethasone induced thymic apoptosis [19] and cancer [18, 20].

The aim of the current study was to create a bifunctional brain-penetrant fluorescent (Oregon Green) and PET-detectable tracer molecule [^68^Ga]Ga-TC3-OGDOTA designed to accumulate in vivo in cells with increased levels of CC3 activity. We have recently shown that it is possible to target cathepsin D in vivo in Alzheimer's disease (AD) using a different tracer with a similar design [21–23]. These tracers contain an HIV1 tat-based CPP, which allows bidirectional transport of the molecule both into and out of cells and, thus, helps the tracer cross the blood brain barrier and cell membranes. Inside an apoptotic cell, activated caspases cleave the DEVD peptide, releasing the CPP and trapping the imaging moiety inside the cell. We hypothesized that [^68^Ga]Ga-TC3-OGDOTA accumulation would increase in the brain of mouse models of stroke and AD and that [^68^Ga]Ga-TC3-OGDOTA would specifically label apoptotic cells.

## 2. Materials and Methods

### 2.1. Design of the Tracer Targeting Cells with Increased CC3 Activity

**Figure 1:**
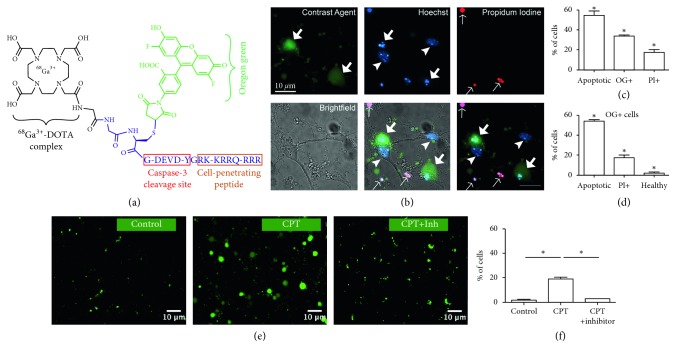
Ga-TC3-OGDOTA accumulates in apoptotic cells. (a) Design of [^68^Ga]Ga-TC3-OGDOTA. (b–f) Ga-TC3-OGDOTA (green) accumulation in neuronal cell cultures treated with CPT. (b) Ga-TC3-OGDOTA predominantly accumulated in cells showing apoptosis by characteristic nuclear morphology (DAPI, blue); minor Ga-TC3-OGDOTA staining was also detected in dead cells, positive for PI (red). Thick arrows point to apoptotic cells, arrowheads to healthy cells, and thin arrows to dead cells. (c) Quantification of cells positive for apoptotic nuclear morphology, Ga-TC3-OGDOTA, and PI. (d) Distribution of Ga-TC3-OGDOTA cells (OG+) within apoptotic, dead, and healthy neurons. (e, f) Ga-TC3-OGDOTA fluorescence in cultures treated with CPT or CPT and an apoptosis inhibitor Z-DEVD-FM. (e) Representative images for each treatment. (f) Quantification of Ga-TC3-OGDOTA-positive cells.

To design a tracer targeting apoptotic cells, we followed a strategy analogous to the one used in our previous studies of a cathepsin D-targeting tracer/contrast agent [21–24], but replacing the cathepsin D cleavage sequence with that of CC3 (Figure 1(a)). In the resulting [^68^Ga]Ga-TC3-OGDOTA, the HIV TAT CPP is linked to a PET label, DOTA molecule complexing ^68^Ga^3+^, and a fluorescent Oregon Green label using the CC3-cleavable peptide DEVD. The TAT peptide allows transport of the agent into and out of the cell. It has been shown previously that caspase-3 readily cleaves similar peptide sequences on the carboxy side of the second aspartic acid residue [13, 15, 25]. When cleaved within the cell cytoplasm by CC3, the CPP is free to diffuse out of the cell and the non-cell-permeating fluorescent and radioactive labels are expected to be retained inside the cell (Supplementary Figure 1(a)).

### 2.2. Tracer Synthesis

The PET/fluorescence-labeled tracer targeting activated (cleaved) caspase-3 [^68^Ga]Ga-TC3-OGDOTA was prepared using the method previously described in detail for a cathepsin D-targeted contrast agent [21–24] by replacing the cathepsin D cleavage peptide sequence with the DEVD peptide. Reagents were commercially available, and all solvents were HPLC or peptide synthesis grade (Caledon Laboratories) except for water (18.2 MΩ·cm Millipore water), and aqueous solutions were lyophilized.

### 2.3. General Procedures

Oligopeptides used in this study were assembled on a peptide synthesizer (CEM Liberty Blue) using standard solid phase peptide synthesis (SPPS) protocols. Rink Amide MBHA resin (100–200 mesh; Peptides International) was used as well as the protected amino acid monomers D-*t*Bu, E-*t*Bu, R-Pbf, Q-Trt, K-Boc, Y-*t*Bu, C-Trt (N-terminal-Fmoc; Peptides International). Ultraperformance liquid chromatography (Method A) was performed on a BEH C18 column (particle size 1.7 *μ*m; 1.0 id × 100 mm) using a chromatograph (Waters Acquity UPLC) equipped with an autoinjector, an HR-ESI-MS detector (Waters-Micromass, LCT Premier XT), and a photodiode array UV detector 990M (Waters). The mobile phase was gradient from 100% H_2_O to 100% MeCN (both solvents containing 0.1% HCOOH) over 5 min, then 100% MeCN over 2 min, with flow rate of 0.1 mL/min. HPLC purification (Methods B and C) was performed on a Delta-Pak C_18_ 300 Ǻ column (particle size 15 μm; 8 × 100 mm Radial-Pak cartridge) using a chromatograph (Waters Delta 600) equipped with an in-line filter, an autoinjector, a fraction collector, and a photodiode array UV detector 996 (Waters). The mobile phase for Method B was a gradient from 99% H_2_O/1% MeCN to 90% H_2_O/10% MeCN in 10 min, then to 85% H_2_O/15% MeCN in 11 min, at flow rate of 3 mL/min. The mobile phase for Method C was gradient from 90% H_2_O/10% MeCN to 100% MeCN in 10 min, at flow rate of 3 mL/min. Verification of radiolabeled conjugate purity was carried out by analytical radio-RP-HPLC (Sunfire^TM^ C_18_ 300 Å column; particle size 15 *μ*m; 8 × 100 mm) using a Waters 1525 Binary HPLC pump containing a dual *λ* absorbance detector (Waters 2487), in-line degasser, a gamma detector Model 105S (Carroll and Ramsey Associates), and Breeze software (version 3.30) using a gradient from 70% H_2_O/30% MeCN to 100% MeCN in 10 min, at flow rate of 1 mL/min (Method D). All solvents in Methods B, C, and D contained 0.1% TFA. Mass spectra (MS) were obtained on a mass spectrometer (Micromass LCT Premier XT) using electrospray ionisation. Radiolabeling was performed with multiple runs (*n* = 13) on a fully automated ^68^Ge/^68^Ga synthesizer Modular-Lab (Eckert & Ziegler). 4-(2-Hydroxyethyl)-1-piperazineethanesulfonic acid (HEPES) buffer (1 M) used for radiolabeling was prepared by dissolving HEPES (23.8 g, 100 mmol) in water (100 mL), followed by adjusting the pH to 3.50 using 12 M HCl solution.

### 2.4. Preparation of DOTA-Peptide Conjugate for Radiolabeling

**Scheme 1:**
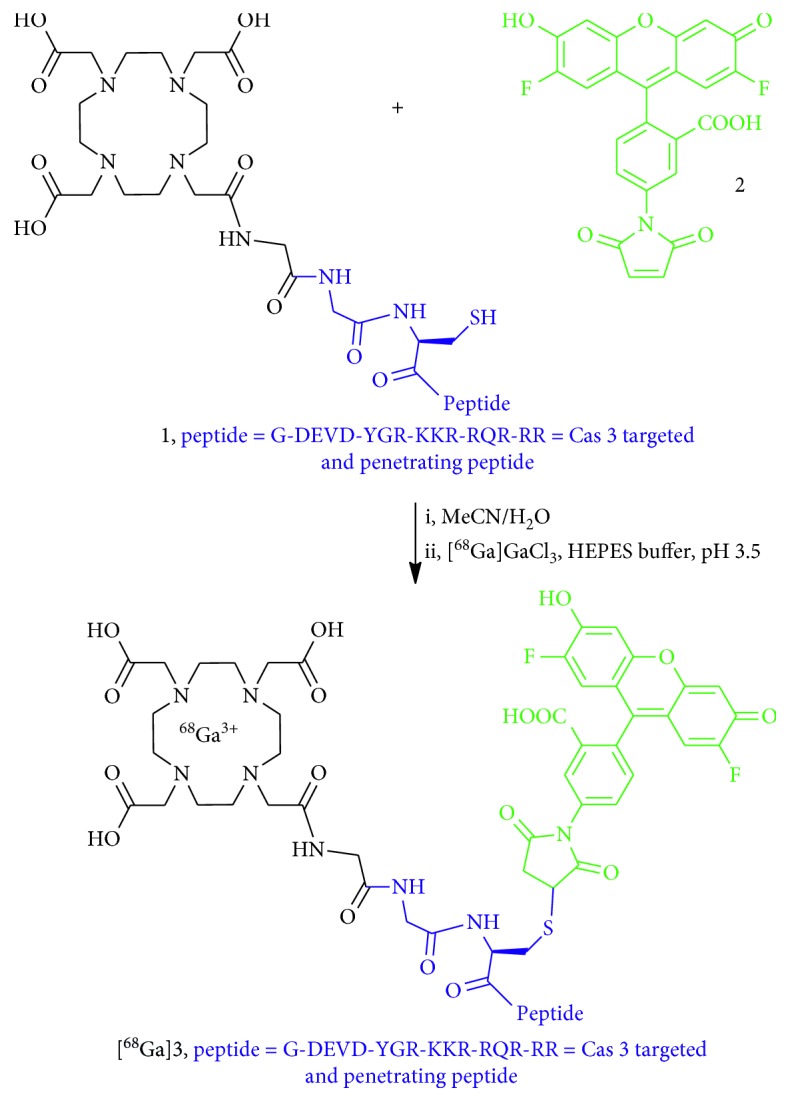
Preparation of radiolabeled probe [^68^Ga]**3** = [^68^Ga]Ga-TC3-OGDOTA.

Conjugation of 1,4,7,10-tetraazacyclododecane-1,4,7,10-tetraacetic acid (DOTA) terminal monomer with a peptide on the resin was carried out as described previously (scheme 1, [24]). Crude conjugate (**1**) was purified by semipreparative HPLC as described in general experimental protocols, Method C. The pure conjugate (**1**) was obtained as a trifluoroacetate salt from lyophilization of HPLC fractions (9.6 mg, 5%, based on 0.05 mmol scale used for the SPPS), colorless solid, HPLC (Method C): *t*_R_ 4.2 min; HRMS (ESI) *m/z*: found 2677.3674 [M+H]^+^ (2677.3691 calcd). Treatment of (**1**) (9.6 mg, 2.3 *μ*mol) with Oregon Green [(**2**), 1 mg, 2.3 *μ*mol, Thermo Fisher] was carried out as described previously [24]. The pure fluorophore (Oregon Green) conjugate was obtained as the trifluoroacetate salt (4.7 mg, 46%), red solid (Method C): *t*_R_ 5.1 min; HRMS (ESI) *m/z*: found 3141.4236 [M+H]^+^ (3141.4273 calcd).

### 2.5. Metallation of DOTA-Peptide Conjugate with GaCl_3_

The fluorophore-peptide-chelator conjugate (0.4 mg, 9 nmol) was dissolved in an acetate buffer (100 *μ*L, pH 3.5), followed by the addition of GaCl_3_ (Strem Chemicals) solution in water (2 mM, 90 *μ*L). The mixture was incubated for 20 min at 80°C; the completion of the reaction was verified by mass spectrometry. The mixture was transferred to a centrifuge tube, frozen and lyophilized. The residue was used as a “cold” standard Ga-TC3-OGDOTA for radiotracer [^68^Ga]Ga-TC3-OGDOTA (**3**) without further purification, HPLC (Method C): *t*_R_ 2.69 min; HRMS (ESI) *m/z*: found 3207.3138 [M-2H]^+^ (3207.3294 calcd).

#### 2.6. Preparation of Radiolabeled Conjugate [^68^Ga]Ga-TC3-OGDOTA

Oregon Green conjugate (0.03 mg, 6.7 nmol, (**2**) on Scheme 1) was dissolved in water (55 *μ*L) and added to 700 *μ*L, 1 M HEPES, pH 3.5, in a sterile vial (Scheme 1). Radiolabeling was performed as described in General procedures. A solution of [^68^Ga]GaCl_3_ in 0.1 M HCl was eluted into the vial to reach the activity 219.2 ± 22.1 MBq. The vial was incubated for 10 min at 100°C, followed by solid phase extraction on a Waters light tC18 Sep-pak cartridge, consecutively eluting with water (2 mL) and EtOH (2.5 mL). The radiolabeled peptide ([^68^Ga]**3**, Scheme 1) was obtained as a solution in EtOH (in a sterile vial), the activity of the peptide solution was 41.7 ± 10.1 MBq, radiochemical yield (decay corrected after 15 min, 10 min synthesis +5 min drying) 19 ± 4%, molar activity 6.22 ± 1.51 GBq/μmol. EtOH was removed by a stream of N_2_ at 80°C; the residue was subsequently used for the animal studies. The purity of radiotracer (**3)** was verified by HPLC (Method C, *t*_R_ 2.58 min), by comparison with the chromatograms corresponding to the “cold” standard described above (Supplementary Figure 1(b)). The absence of colloidal ^68^Ga was ensured by the solid phase extraction step as described elsewhere [26] and supported by nonaugmented tracer uptake (Supplementary Figure 3(b)) in liver (0.78% ID/g) and bone marrow (hip, 0.07% ID/g).

#### 2.7. In Vitro Validation: Primary Neuronal Cultures

For the in vitro cell culture studies, primary hippocampal and cortical neuronal cultures were prepared from E17 mouse embryos as described previously [27] and cultured in regular neuronal medium (RNM) containing 1x B27, 0.5 mM glutamine, 0.25% glucose in Neurobasal medium (Invitrogen). Both male and female embryos were used for cultures as the toxicity conditions used in this study have not been shown to depend on sex differences.

Three different conditions known to induce neuronal apoptosis were used to study Ga-TC3-OGDOTA uptake in cell culture: camptothecin (CPT) toxicity, OGD, and A*β*O toxicity. These assays model different conditions that may occur in vivo and demonstrate the versatility of the tracer. For the CPT toxicity assay, cortical neurons were treated with 0 (control) or 10 *μ*M CPT (Sigma-Aldrich) for 24 h at normal conditions (5% CO_2_, 37°C). After that the cultures were washed with RNM and incubated with various concentrations of Ga-TC3-OGDOTA for 7 h, followed by either direct live imaging or fixation with 4% paraformaldehyde (PFA) in PBS for staining.

For OGD studies, the media in the hippocampal neuronal cultures on day 7 were saved and replaced with RNM without glucose. Cells were incubated for 1 h at 37°C in 95% N_2_/5% CO_2_ atmosphere with less than 0.2% O_2_ as described previously [28]. For control experiments, cells were incubated for the same time under normal conditions. 1-hour OGD was followed by 7-hour reperfusion in the saved media supplemented with 3 *µ*M Ga-TC3-OGDOTA under normal conditions. After that, cultures were either imaged live directly or fixed with 4% PFA for staining.

For the A*β*O toxicity assay, A*β*Os were prepared from A*β* (1–42) monomer (rPeptide, cat#A-1002-2) as described previously [27]. The quality of the A*β*O preparations was checked by Western blotting as described previously [27], using Bolt 4–12% precast gels (Thermo Fisher, cat#NW04120BOX). 1 µM A*β*Os (or vehicle for controls) was added to hippocampal neuronal cultures on day 11. The amount of toxic trimers and tetramers of A*β* estimated by Western blotting was ∼10–20 nM [27]. This is within the range found to be toxic in other studies using synthetic [29] or AD brain-purified [30] A*β*Os. Cell cultures were incubated for 24 h, and 3 *µ*M Ga-TC3-OGDOTA was added for the last 7 h. After that, cultures were either imaged live directly or fixed with 4% PFA for staining.

For live imaging of Oregon Green, cultures were washed 3x with Krebs-Ringer HEPES (KRH) buffer and imaged using an FV1000 confocal microscope (Olympus) equipped with a 20x/0.75 lens with excitation at 488 nm and emission at 505–550 nm.

For coimaging of Hoechst 33342 (Hoechst), 4′,6′-Diamidino-2-Phenylindole (DAPI), Oregon Green and CC3, fixed cultures were immunostained with anti-CC3 antibody (1 : 500, Cell Signaling, cat#9661) followed by antirabbit secondary antibody and DAPI or Hoechst. After that, cultures were mounted on slides and imaged using an FV1000 confocal microscope (Olympus) equipped with a 20x/0.75 objective. Cells positive for tracer, CC3, and total number of cells were counted using the ImageJ (NIH, Bethesda, MD) Cell Counter plugin and their ratio was compared between the treatments.

Cell viability was assessed using the ImageJ Cell Counter plugin. Specifically, the toxicity of the agent was calculated as the percentage of nuclei showing characteristics of dead cells in Hoechst or DAPI stained sections.

#### 2.8. In Vivo and Ex Vivo Validation: Middle Cerebral Artery Occlusion (MCAO) and AD Mouse Models

For in vivo validation of tracer uptake and colocalization with CC3, we used mouse models of MCAO and AD. All procedures were conducted in accordance with the guidelines of the Canadian Council of Animal Care and approved by the University of Western Ontario Animal Use Subcommittee (protocol 2016–103 and 2016–104). The study also complied with the ARRIVE guidelines (Animal Research: Reporting in Vivo Experiments). To test whether [^68^Ga]Ga-TC3-OGDOTA could detect brain injury/apoptosis in vivo, we employed a widely used model of stroke, MCAO. During this procedure, blood flow is blocked resulting in ischemic injury predominantly in the striatum and cortex, which during the ensuing reperfusion/recovery period can also affect adjacent brain regions [31, 32] (Supplementary Figure 3(a)). MCAO is characterized by an extensive core, or edema, consisting of massive necrotic tissue, and by a penumbra, which is present at the periphery of the core and is characterized by abundant apoptosis [33]. MCAO was performed as previously described [28] in 3–4 month old wild-type male mice (weight, average ± SD: 28 ± 2 g) in either C57BL/6J or B6SJL background. Briefly, isoflurane-anaesthetized mice were immobilized under the operating microscope, and a 0.06–0.09 mm nylon suture was inserted via the left carotid artery into the internal carotid artery and then into the circle of Willis to occlude the MCA. Sham-operated mice were subject to the same procedure except for the suture-induced occlusion. Mice were then removed from anaesthesia and kept under a heat lamp for 60 min, after which they were anaesthetized again and the suture was removed. Operated mice were held in a cage with a heated pad for 24 h, receiving 1 mL saline injections i.p. every 12 h. Neurological state was assessed using the 5-point Bederson scale and only mice scoring 2 (severe forelimb weakness, consistent turns to the deficit side when lifted by the tail) or 3 (compulsory circling) were used for the experiments. MCAO efficiency was estimated by triphenyl tetrazolium chloride (TTC) staining. After 24 h of reperfusion, the animals were euthanized. Brains were frozen at –80°C for 15 min and sliced into 1 mm coronal sections. The brain slices were immersed in the 2% TTC solution for 15 min at 37°C, washed with 0.9% saline solution, and imaged by a digital camera. The ischemic damage to coronal brain sections was qualified by the absence of staining.

In AD, neurodegeneration eventually leads to the shrinkage of the hippocampus and cortex, but in vitro and ex vivo experiments suggest that neuronal apoptosis can start as early as the accumulation of A*β* [34, 35]. Neurodegeneration is not frequently observed in mouse models of AD, and the 5xFAD model is one of the few AD mouse models that present this AD hallmark [36]. Neurodegeneration, including neuronal loss, and increased caspase-3 activity were shown to manifest by the age of 8–9 months; therefore, we used 5xFAD mice older than 8 months to test [^68^Ga]Ga-TC3-OGDOTA. 5xFAD mice in B6SJL background (B6SJL-Tg(APPSwFlLon, PSEN1∗M146L∗L286V)6799Vas/J) were purchased from Jackson Laboratories (Bar Harbor, ME) and cross-bred with wild-type B6SJL mice in our facility. Both male (weight, average ± SD: wild-type- 34 ± 7 g, transgenic- 32 ± 4 g) and female (weight, average ± SD: wild-type- 29 ± 7 g, transgenic- 22 ± 3 g) littermates were used for the experiments. A summary of animals used for this work is provided in Table 1.

#### 2.9. Positron Emission Tomography (PET) in Mice

Imaging was performed as described previously for the cathepsin D tracer [22], using list mode scanning on the Inveon preclinical PET system (Siemens). PET scanning and data analysis using the CARIMAS software (Turku PET centre, Finland) were performed following the standard procedures. All mice received a slow intravenous injection of 11.2 ± 1.0 MBq [^68^Ga]Ga-TC3-OGDOTA suspended in ∼150 *µ*L saline through a tail vein catheter. To reduce discomfort, the solution was adjusted to a nearly neutral pH using dropwise dilutions of HCL/NaOH and litmus paper prior to administration. Imaging was initiated ∼1 min before injection of the tracer and lasted for 2 h. Histograms were built using dynamic step intervals set to 5 × 30 s at the beginning of the imaging, followed by 5 × 60 s, 5 × 300 s, and 600 s steps for the remaining time. Acquisition (energy window 350–650 keV, timing window 3.432 ns), histograms, and reconstructions were performed using the Siemens Inveon software supplied with the scanner. Histograms were built using separate delay handling, global deadtime correction, and 79 ring difference for maximum sensitivity. For reconstruction, the Ordered-Subsets Expectation Maximization (OSEM3D) with ordinary Poisson Maximum a posteriori OP-MAP (2 OSEM3D iterations, 18 MAP iterations) algorithm was used. The resulting 3D data set had a matrix of 128 × 128 × 159 and a voxel size of 0.78 × 0.78 × 0.80 mm in the *x*-, *y*-, and *z*-directions. Attenuation correction was not performed as the mouse head size does not justify the use of such a correction and does not vary much between animals. Animals were kept warm (∼37°C) using a floor mounted heating lamp before, during, and immediately after the imaging session with body temperature monitored by rectal probe. For scanning, mice were anaesthetized with isofluorane (<1.5%). For brain fluorescence imaging following MCAO, mice were kept on the heating pad until their euthanasia. Mice were studied at 3-4 months (MCAO, *N* = 5; sham *N* = 3), ∼8.5 months (male: 5xFAD, *N* = 3; wild type, *N* = 3), and 11 months (female: 5xFAD, *N* = 7; wild type, *N* = 6) of age.

#### 2.10. PET Data Analysis

**Figure 2:**
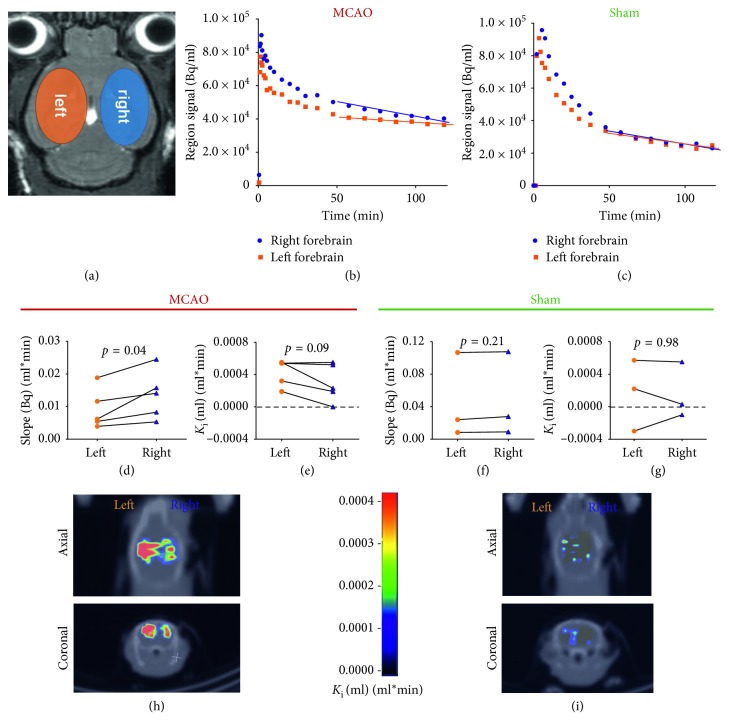
PET kinetics of [^68^Ga]Ga-TC3-OGDOTA retention in MCAO and sham-operated mouse brains. (a) Scheme of ROIs for PET signal integration over the left (occluded) and right (nonoccluded) brain halves. (b–i) PET signal kinetics and their analyses in MCAO and sham-operated brains. (b) Sample PET kinetics in MCAO brain halves showing linear approximation during the 2nd hour of signal acquisition. (c) The same for sham-operated brains. (d, e, h) Comparison of linear approximation slope (d) and graphic analysis Patlak constants (e) for MCAO brains. (h) Representative coronal and axial sections of *K*_i_ mapping in an MCAO mouse brain. *K*_i_ map was aligned with a CT mouse image manually as described in Methods. (f, g, i) The same as (d, e, h) but for sham-operated brains. Data were collected from 5 MCAO and 3 sham-operated mice.

**Figure 3:**
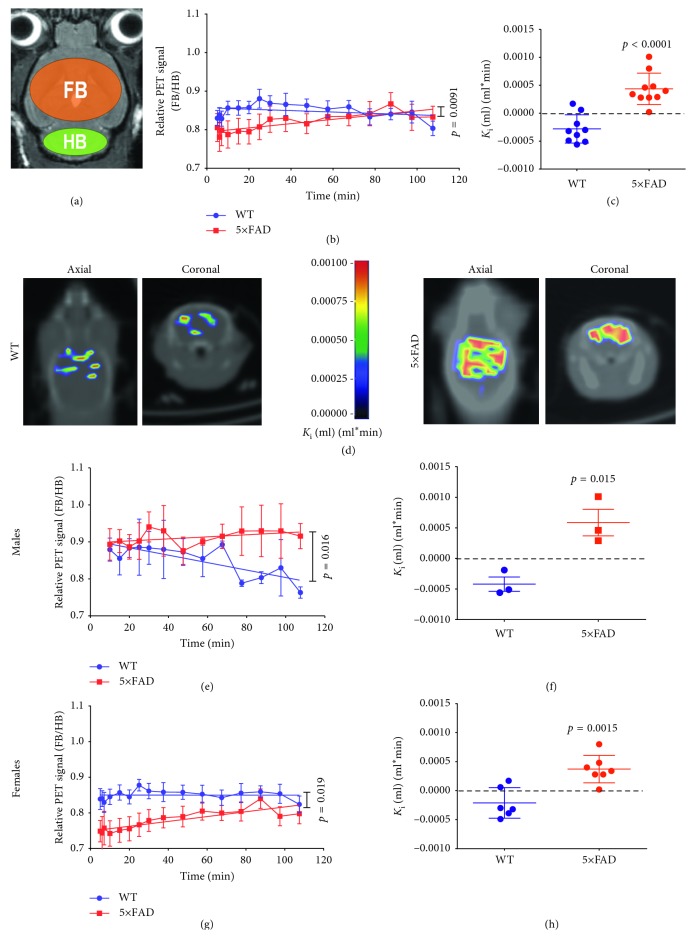
PET kinetics of [^68^Ga]Ga-TC3-OGDOTA retention in 5xFAD mouse brains and wild-type controls. (a) Scheme of ROIs for PET signal integration over the forebrain (fb) and hindbrain (hb, control area). (b–h) Relative (fb/hb) PET signal kinetics and their analyses in 5xFAD mouse brains compared to wild-type controls. (b) Relative PET kinetics in 5xFAD and control 8-month-old male brains were linearly approximated starting from ∼10 min timepoint. (c) Comparison of Patlak *K*_i_ for (b) kinetics. (d) Representative coronal and axial sections of *K*_i_ mapping in a wild-type (WT, left) and a 5xFAD (right) mouse brains. The *K*_i_ map was aligned with a CT mouse image manually as described in Methods. (e, g) Relative PET kinetics in 5xFAD and control male (e) and female (g) brains were linearly approximated starting from ∼10 min timepoint. (f) Comparison of Patlak *K*_i_ for (e) kinetics. (h) Comparison of Patlak *K*_i_ for (g) kinetics.

The pattern of tracer uptake and washout from brain and other mouse organs (Supplementary Figure 3(b)) was as expected with a rapid increase followed by a gradual washout from the brain. Analysis of the linear decay of the PET signal kinetics as well as Patlak graphical analysis were used to study [^68^Ga]Ga-TC3-OGDOTA uptake in mouse brains. Region of interest (ROI) assignment and Patlak graphical analysis [37, 38] of the time-activity curves (TACs) derived from these ROIs were performed using the CARIMAS software (Turku PET centre, Finland) using the embedded Patlak function. As a reference region, the left ventricle was used in the stroke mouse model and the hindbrain in the AD mouse model. PET signals from the [^68^Ga]Ga-TC3-OGDOTA injected into MCAO and sham-operated mice were analyzed by comparing operated (ipsilateral) vs. nonoperated (contralateral) sides of forebrain. We assumed that in an MCAO brain, the ipsilateral ROI would contain most of the MCAO-induced damaged tissue, while the contralateral ROI would contain healthy tissue (Figure 2(a)). We normalized PET signal in AD-affected forebrain with that in hindbrain (Figure 3(a)), a brain region that exhibits significantly lower beta-amyloid production [39] and accumulation [22] and has been successfully used as a control ROI in previous PET studies of 5xFAD mice [22, 40]. To facilitate ROI assignment and distinguish brain from surrounding tissue, the integrated PET signal was manually aligned with a computed tomography (CT) image of a mouse of the same genetic background and similar weight, as described previously [22]. The CT examination used for visual guidance of anatomical landmarks was performed with the Revolution CT (GE HealthCare Ltd.) human scanner using a helical scan of 120 kVp with a 1.25 mm slice thickness and a 0.2 × 0.2 mm in-plane resolution. Representative Patlak *K*_i_ maps for each condition or genotype were prepared by the CARIMAS-embedded image filter function and manually aligned with a mouse CT image. ROI-derived TACs were also exported into GraphPad Prism 6.0 software and compared using 2-way analysis of variance (ANOVA) and linear approximation.

#### 2.11. Confocal Microscopy on 20 µm Brain Sections

20 h after [^68^Ga]Ga-TC3-OGDOTA injection, mice were anaesthetized and transcardially perfused with PBS followed by 4% PFA in PBS as described previously [41]. Immunohistochemistry on brain sections was performed as described previously [42], using rabbit anti-CC3 antibody (1 : 500), antirabbit Alexa633 secondary antibody, and Hoechst 33342. Sections were imaged with an FV1000 confocal microscope equipped with 10x/0.4 and 20x/0.75 objectives. Obtained images were analyzed using ImageJ.

#### 2.12. Optical Clearing of Hydrogel-Embedded Mouse Brains

For confocal imaging of 2 mm brain sections mice were anaesthetized 20 h after [^68^Ga]Ga-TC3-OGDOTA injection, placed on ice and transcardially perfused with hydrogel monomer solution (4% PFA, 4% acrylamide, 0.05% bis-acrylamide, 1 g/L VA-044 (Wako Chemicals USA, VA) in PBS) as described for the CLARITY procedure [43]. After 48-hour gel polymerization the brains were cut with VT1200 vibratome (Leica) into 2 mm sections, which were then cleared under constant flow of 4% SDS in 0.2 M sodium borate, pH 8.5, for 72–96 h. In order to visualize the sites of accumulation of [^68^Ga]Ga-TC3-OGDOTA in the AD mouse model, we used optical clearing of [^68^Ga]Ga-TC3-OGDOTA-injected 5xFAD and control brains.

#### 2.13. Confocal Microscopy on 2 mm Thick Mouse Brain Sections

Optically cleared sections were placed into SeeDB mounting medium (80.5% w/w D-fructose, 0.5% thioglycerol, [44]) and imaged using an FV1000 confocal microscope equipped with air 10x/0.4, 20x/0.75, or silicon oil 30x/1.05 objectives.

For CC3 imaging, the tissue was incubated with anti-CC3 antibody (1 : 50) in PBS-T for 5 days at 37°C, followed by washing with PBS-T 3 × 12 h, incubation with anti-rabbit Alexa Fluor 633 secondary antibody (1 : 50) in PBS-T for 3 days at 37°C and washing with PBS-T 3 × 12 h. After that, the sections were imaged in SeeDB mounting medium using the same excitation/emission parameters as for thin brain sections. 3D reconstruction and image analysis were performed using ImageJ 3D stack module.

### 2.14. Statistics

For statistical analysis of fluorescence, Student's *t*-tests (paired or unpaired, when needed) were performed using GraphPad Prism 6 software. For statistical analysis of PET kinetics, linear approximation, Student's *t*-test, and 2-way ANOVA were performed using GraphPad Prism 6, and Patlak analysis [37] was performed using the CARIMAS software. Power and sample size analysis based on the PET data obtained in this study was performed using the Matlab function “sampsizepwr” (https://www.mathworks.com/help/stats/sampsizepwr.html, MathWorks).

## 3. Results

### 3.1. Ga-TC3-OGDOTA Accumulates in the CPT Cell Culture Model

Treatment of primary cortical cultures with the DNA topoisomerase I inhibitor CPT, shown previously to induce neuronal apoptosis [45], elevated the number of apoptotic neurons in the culture (Supplementary Figures 2(a) and 2(b)). When Ga-TC3-OGDOTA was added to CPT-treated cultures, it accumulated predominantly (88%) in the cells showing characteristic apoptotic nuclear morphology, with only minor nonspecific staining of the dish surface and dead cells (Figures 1(b)–1(d)). This increase in Ga-TC3-OGDOTA-positive cells was prevented when neuronal cultures were cotreated with CPT and the CC3 inhibitor Z-DEVD-FM (Figures 1(e) and 1(f)), which also decreased apoptosis-related changes in nuclear morphology.

### 3.2. TC3-OGDOTA Accumulates in Stroke and AD Cell Culture Models

**Figure 4:**
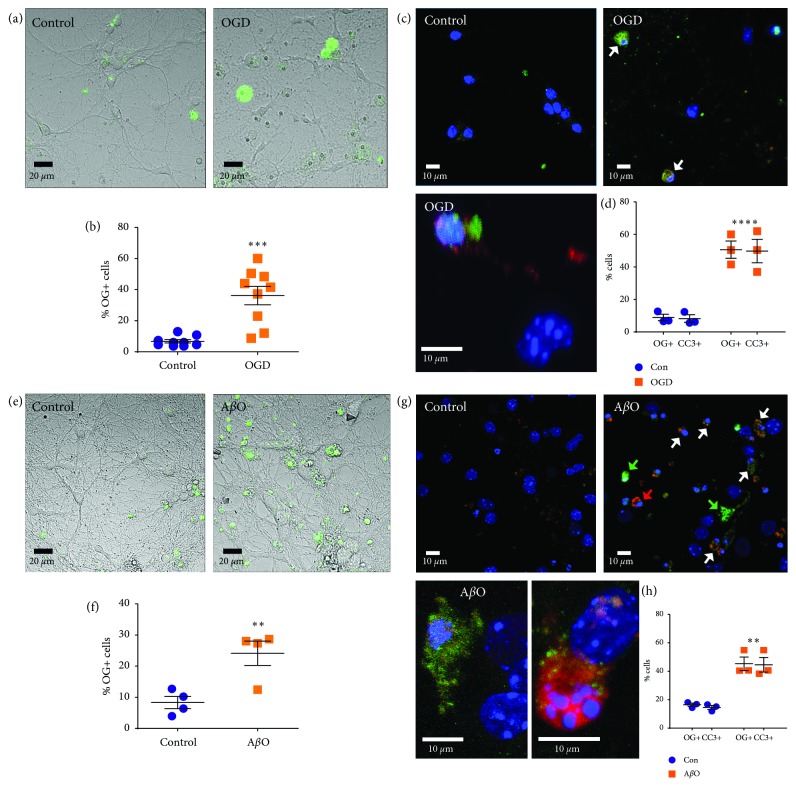
Ga-TC3-OGDOTA accumulates in apoptotic neurons in vitro in ischemia and AD cell culture models. (a–d) Ga-TC3-OGDOTA accumulation in primary neurons treated with OGD was analyzed in live cultures (a, b) and in fixed cultures costained for nuclei (Hoechst) and CC3 (c, d). (a) Representative images of live OGD-treated and control cultures. (b) Quantification of Ga-TC3-OGDOTA-positive (OG+) live cells in OGD/control cultures. (c) Representative images of fixed/immunostained control (top-left), OGD (top-right), and OGD zoomed (bottom) cultures. (d) Quantification of Ga-TC3-OGDOTA-positive (OG+) and CC3-positive cells in fixed OGD/control cultures. (e–h) Ga-TC3-OGDOTA accumulation in primary neurons treated with A*β*Os was analyzed in live cultures (e, f) and in fixed cultures costained for nuclei (Hoechst) and CC3 (g, h). (e) Representative images of live A*β*O-treated and control cultures. (f) Quantification of Ga-TC3-OGDOTA-positive (OG+) live cells. (g) Representative images of fixed control (top-left), A*β*O (top-right), and A*β*O zoomed (bottom) cultures. (h) Quantification of Ga-TC3-OGDOTA-positive (OG+) and CC3-positive cells in fixed A*β*O/control cultures. Cells presenting high levels of both Ga-TC3-OGDOTA and CC3 are marked with white arrows, while those with preferential Ga-TC3-OGDOTA or CC3 accumulation are marked with green or red arrows, respectively. Data were collected from at least 3 independent cultures for each condition and analyzed with Student's *t*-test or Two-way ANOVA. ^*∗*^ *p* < 0.05; ^*∗∗∗*^ *p* < 0.001; ^*∗∗∗∗*^ *p* < 0.0001.

Analysis of the OGD-treated ischemic cultures showed an increased number of neurons that were both positive for CC3 and apoptosis-related changes in nuclear morphology, as detected by the Hoechst fluorescent dye (Supplementary Figure 2(c)). When added to the media during post-OGD incubation under normal conditions, Ga-TC3-OGDOTA accumulated in 40% of neurons, compared to only ∼5% accumulation in nonischemic controls (Figures 4(a) and 4(b)). As with CPT treatment, intracellular localization of Ga-TC3-OGDOTA was ubiquitous throughout the cell (Figure 4(a)). Immunostaining of these cultures for CC3 revealed a very similar increase in the proportion of cells positive for Ga-TC3-OGDOTA and CC3 under both OGD and control conditions (Figures 4(c) and 4(d)). Notably, almost all (91%) of the cells positive for either Ga-TC3-OGDOTA or CC3 had both markers present. Intracellular localization of Ga-TC3-OGDOTA after immunostaining differed to some extent from that in live cultures, probably due to fixation and partial washout.

24-hour A*β*O treatment induced significant apoptosis in neurons (Supplementary Figure 2(d)) and fluorescence imaging in live cultures revealed an increase in Ga-TC3-OGDOTA-positive neurons, similar to that observed with CPT and OGD treatments (Figures 4(e) and 4(f)). Moreover, Ga-TC3-OGDOTA was seen predominantly (∼90%) in neurons presenting CC3 staining and apoptotic nuclear morphology (Figures 4(g) and 4(h)), similar to the OGD experiment. In fixed OGD and A*β*O-treated cultures, Ga-TC3-OGDOTA was distributed ubiquitously in some cells, similarly to the pattern observed in live cultures. At the same time in other cells, the tracer appeared to have a compartment/aggregate-like localization (Figures 4(c) and 4(g)). While Ga-TC3-OGDOTA and CC3 immunoreactivity were generally colocalized within a cell (Figures 4(c) and 4(g)), some cells exhibited preference towards one or the other dye (Figure 4(g), bottom panel).

It is noteworthy that in both neuronal culture models of apoptosis, Ga-TC3-OGDOTA was not cytotoxic at the concentrations studied. In control cultures incubated with Ga-TC3-OGDOTA, 17 ± 1% of cells showed evidence of neuronal damage, which was similar to that observed in control cultures without Ga-TC3-OGDOTA (16 ± 2%). This observation corroborates our previous experiments with a similar compound using TAT [23].

### 3.3. PET Detects [^68^Ga]Ga-TC3-OGDOTA Retained by Ischemic Brains in Mice

After the initial exponential drop, the PET signal showed almost linear kinetics starting at ∼50 min (Figures 2(b) and 2(c)). In the MCAO brains, the PET signal decayed more slowly on the ipsilateral side compared to the contralateral, suggesting accumulation of [^68^Ga]Ga-TC3-OGDOTA in the ischemic tissue (Figure 2(b)). Lateralized differences in PET signal dynamics were not observed in sham-operated controls (Figure 2(c)). Indeed, the linear approximation of the second hour of the kinetics showed significantly lower decay constant on the occluded side, but only in MCAO brains (Figures 2(d) and 2(f)). We also employed Patlak graphical analysis [37, 38], which can show irreversible binding of the ligand in the selected region. We used the heart as the reference region representing zero retention (http://www.turkupetcentre.net/petanalysis/model_reference_tissue.html). In MCAO (Figures 2(e) and 2(h)), but not sham-operated (Figures 2(g) and 2(i)) mice the retention constant (*K*_i_) on the side ipsilateral to the surgery side of the forebrain was positive (*p*=0.004), suggesting [^68^Ga]Ga-TC3-OGDOTA accumulation in MCAO, but not in sham-operated brains. Interestingly, in MCAO brains, *K*_i_ was generally positive even on the contralateral side (*p*=0.042, Figure 2(e)), with a tendency to be smaller than on the ipsilateral side (*p*=0.090). This result suggests that the side contralateral to MCAO may also have accumulated [^68^Ga]Ga-TC3-OGDOTA. It is possible that brain damage in some cases might have spread into the contralateral half of the brain, which has been observed previously in MCAO mouse models [46, 47]. Variations in the severity of the injury lead to poor definition of the ROI, which together with changes in cerebral blood circulation in the infarct and penumbra areas could explain the failure to distinguish ipsilateral and contralateral sides by Patlak analysis.

### 3.4. Cells on the Ischemic Side Display [^68^Ga]Ga-TC3-OGDOTA Fluorescence

**Figure 5:**
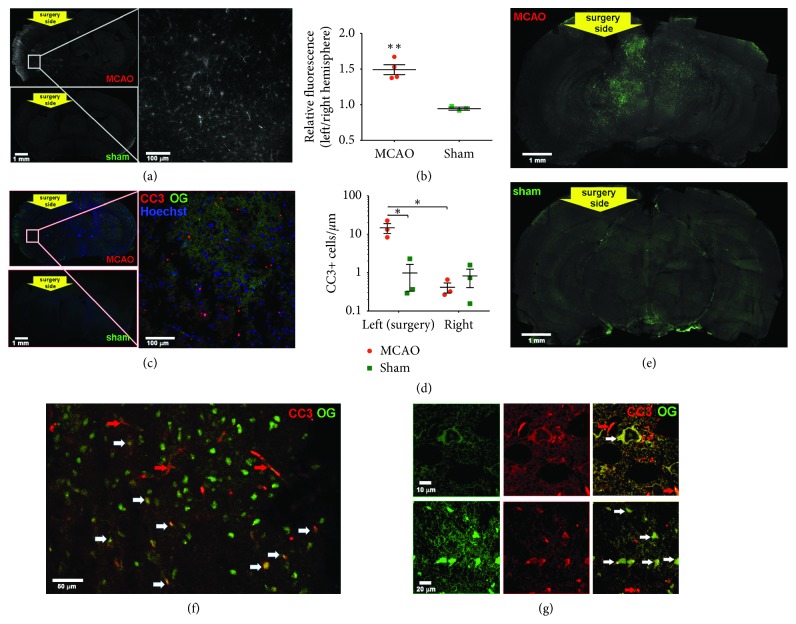
Fluorescence microscopy in brains extracted from [^68^Ga]Ga-TC3-OGDOTA-injected MCAO and sham-operated mice. (a, b) [^68^Ga]Ga-TC3-OGDOTA fluorescence in 20-*µ*m coronal brain sections. (a) Representative images of MCAO (top left and right panels) and sham-operated (bottom left panel) brains. (b) Quantification of fluorescence integrated over the left (operated) vs. right (nonoperated) brain halves. Data were averaged over 4 sections for each brain collected from 4 MCAO and 3 sham-operated mice and analyzed by Student's *t*-test. (c, d) Sections of the same brains immunostained for CC3 (red) and Hoechst (blue). [^68^Ga]Ga-TC3-OGDOTA presence was probed in Oregon Green channel (OG, green). (c) Representative images of MCAO (top left and right panels) and sham-operated (bottom left panel) brains. (d) Quantification of CC3-positive cells in the left (operated) vs. right (nonoperated) brain halves. Data were averaged over 4 sections collected from 3 brains for each condition and analyzed by two-way ANOVA followed by Bonferoni post hoc test. ^*∗*^ *p* < 0.05; ^*∗∗*^ *p* < 0.01 (E-G) [^68^Ga]Ga-TC3-OGDOTA fluorescence (OG, green) in optically cleared 2 mm brains sections. (e) Representative images of 1 mm z-stack images of coronal sections of MCAO (top) and sham-operated (bottom) brains. (f, g) Representative images of the MCAO brain sections immunostained for CC3 and imaged for both [^68^Ga]Ga-TC3-OGDOTA and CC3 (red). Cells positive for both [^68^Ga]Ga-TC3-OGDOTA and CC3 are marked with white arrows, blood vessels—with red arrows. (f) Thalamus, 20x objective, ipsilateral side. (g) Cortex (top) and thalamus (bottom), 60x objective, ipsilateral side.

Imaging of thin coronal slices of mouse brains perfused and fixed 20 h after [^68^Ga]Ga-TC3-OGDOTA injection showed significantly higher fluorescence on the ischemic side in the MCAO brains compared to the contralateral side and of both sides in sham-operated controls (Figures 5(a) and 5(b)). While the Oregon Green fluorescence was detected throughout the ipsilateral side, it was more intense in the periphery than in the injury core, suggesting that [^68^Ga]Ga-TC3-OGDOTA is retained more in the penumbral area compared to the infarct core. Morphological analysis showed that fluorescent objects were mostly single cells and small blood vessels (Figures 5(a) and 5(b), Supplementary Figure 3(c)). In addition, some sections displayed weak and diffuse fluorescence in the cortex, which probably reflected autofluorescence, as a similar pattern was observed in the MCAO brains that were not injected with [^68^Ga]Ga-TC3-OGDOTA (Supplementary Figure 3(c)).

### 3.5. CLARITY Imaging Shows That [^68^Ga]Ga-TC3-OGDOTA Colocalizes with CC3 in Cells

CC3 immunostaining of thin sections showed a preferential staining of the MCAO-operated side compared to the contralateral side and sham-operated controls (Figures 5(c) and 5(d)). In these sections, [^68^Ga]Ga-TC3-OGDOTA fluorescence was faded, and we could not detect significant colocalization of [^68^Ga]Ga-TC3-OGDOTA and CC3, probably, due to detergent-induced washout.

In order to bypass this limitation of immunostaining, we employed CLARITY, shown to maintain protein content while facilitating tissue penetration by antibodies and allowing imaging with millimeter-range depth [43]. First, we confirmed that extensive washing with 4% SDS buffer did not remove [^68^Ga]Ga-TC3-OGDOTA from hydrogen-embedded cells (Supplementary Figure 3(d)). Cleared brain sections (Supplementary Figure 3(e)) showed a fluorescence pattern similar to that detected in thin sections (Figure 5(e)). The ischemic side of the brain presented higher fluorescence intensity compared to the contralateral side and the sham-operated controls (Figure 5(e)), observed predominantly in the penumbral area, including cortex (Supplementary Video 1) and midbrain (Supplementary Video 2) regions. Distribution of the immunostained CC3 in these sections was similar to that of [^68^Ga]Ga-TC3-OGDOTA, with major CC3 localization in cortex (Supplementary Video 3) and midbrain (Supplementary Video 4). Cells positive for both [^68^Ga]Ga-TC3-OGDOTA and CC3 were abundant throughout these regions though a number of cells contained only one of these markers (Figures 5(f) and 5(g)), which may indicate various stages of apoptosis in those cells.

### 3.6. PET Detects [^68^Ga]Ga-TC3-OGDOTA Accumulation in the Forebrain of 5xFAD Mice

PET normalized signal did not differ significantly between 5xFAD mice and wild-type mice, but its attenuation at 10–110 min after injection was negative in 5xFAD mice, but not in wild-type mice (Figure 3(b)). This result suggests that [^68^Ga]Ga-TC3-OGDOTA accumulated in 5xFAD forebrain relative to hindbrain. The Patlak graphical analysis on the kinetics using hindbrain as the reference region supported this argument showing a positive Patlak *K*_i_ constant for 5xFAD, but not wild-type mice (Figures 3(c) and 3(d)). We also separated mice into two groups, males (*N* = 3 for each genotype) and females (*N*=6 for wild-type, *N* = 7 for 5xFAD). Both male (Figures 3(e) and 3(f)) and female (Figures 3(g) and 3(h)) mice showed increased [^68^Ga]Ga-TC3-OGDOTA accumulation via the decay and the Patlak *K*_i_ constant, compared to their wild-type littermates, although with less power due to the lower *N* values.

### 3.7. CLARITY Reveals [^68^Ga]Ga-TC3-OGDOTA Accumulation in 5xFAD Forebrain

**Figure 6:**
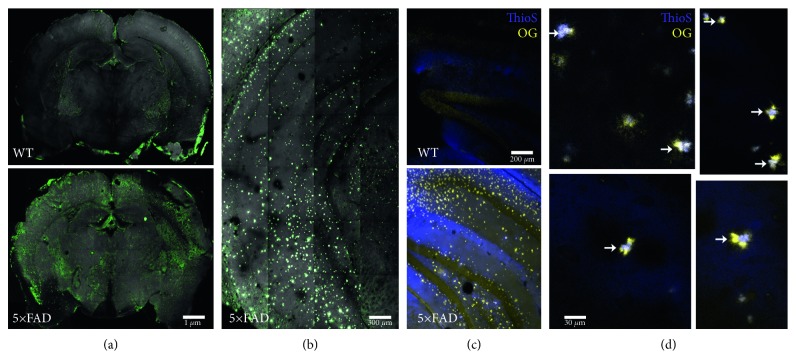
Fluorescence microscopy in optically cleared brains extracted from [^68^Ga]Ga-TC3-OGDOTA-injected 5xFAD and wild-type mice. (a) [^68^Ga]Ga-TC3-OGDOTA fluorescence (green) in optically cleared 2-mm wild-type (top) and 5xFAD (bottom) brain sections. The sections were imaged with 1 mm depth and 3D reconstruction was prepared using ImageJ as described in Methods. (b) [^68^Ga]Ga-TC3-OGDOTA fluorescence in the hippocampus of 5xFAD brains. A coronal section was imaged with 0.3 mm depth and 3D-reconstructed. (c, d) Sections from (a) were stained for amyloid plaques with thioflavin S. (c) Hippocampi of wild-type (top) and 5xFAD (bottom) were imaged for plaques (ThioS, blue) and [^68^Ga]Ga-TC3-OGDOTA (OG, yellow). (d) Confocal images of 5xFAD brains showing amyloid plaques (white arrows) surrounded by [^68^Ga]Ga-TC3-OGDOTA-positive objects.

Confocal imaging showed significantly higher levels of fluorescence in 5xFAD brains compared to wild-type controls (Figure 6(a), Supplementary Video 5). Higher magnification images detected fluorescence coming from both cells and around amyloid plaque-like structures (Figure 6(b), Supplementary Video 6). Indeed, staining with Thioflavin S showed significant colocalization with [^68^Ga]Ga-TC3-OGDOTA throughout the 5xFAD forebrain (Figures 6(c) and 6(d), Supplementary Videos 7 and 8, Thioflavin S(blue), [^68^Ga]Ga-TC3-OGDOTA (yellow)), though [^68^Ga]Ga-TC3-OGDOTA was found more on the periphery of plaques.

## 4. Discussion

In this study, we designed a tracer molecule for PET and fluorescence imaging aimed at identifying cells with increased CC3 activity. We observed in both in vitro and in vivo experiments that its accumulation is directly linked to increased apoptosis. [^68^Ga]Ga-TC3-OGDOTA was detected in cells showing increased levels of CC3 and apoptosis-associated nuclear morphology. In injured brain tissue, it marked the areas previously associated with increased apoptosis such as penumbra in stroke, and cortex and hippocampus in a model of AD. Previous findings, in vitro or ex vivo, showed that ischemic insult [33, 48] or the presence of beta-amyloid oligomers [49, 50] led to apoptotic neuronal death. Similarly, we have shown that these insults significantly increased the number of neurons positive for CC3, a common marker of apoptosis, and that [^68^Ga]Ga-TC3-OGDOTA accumulation follows the same pattern and significantly overlaps with CC3 immunostaining. For these experiments, we used both traditional confocal microscopy on 2D cultures and thin (20-µm) brain sections, and deep-tissue (up to 1 mm) confocal imaging on SDS-cleared hydrogel-embedded 2-mm brain sections. One of the advantages of the latter method is the cross-linking of [^68^Ga]Ga-TC3-OGDOTA amino-groups with the hydrogel mesh, which helps to preserve the tracer in the cells during the clearing/washing steps. Thus, hydrogel-embedding allowed for [^68^Ga]Ga-TC3-OGDOTA coimaging with CC3. Besides, deep-tissue imaging covers a significant portion, if not the whole tissue of interest, and, therefore, is less prone to error.

The current study further advances the imaging of apoptosis in disease. Recently, far-red/near-infrared contrast agents specific to CC3 activity were used in vivo [13, 15] that showed facilitation of the agent delivery into the cell by HIV1 Tat-based CPPs. We also used such CPPs in a tracer/contrast agent targeting cathepsin D, and showed its retention in the 5xFAD forebrain in vivo using PET [22], near-infrared fluorescence [21], and MRI labels [23]. Of note, its accumulation in cells depended on the CPP, justifying the necessity of the CPP presence in the CA structure. Recently, PET ^18^F-labeled tracers, also based on the DEVD peptide have been developed [19, 20]. Future studies should compare the blood-brain barrier penetration efficiency of these agents to our bifunctional tracer.

It is worth noting that in cultured neurons while both Ga-TC3-OGDOTA and anti-CC3 antibody were present in most apoptotic cells, their intensities and subcellular localization differed in some of these cells (Figure 4). One possible explanation is a delayed effect of the tracer accumulation. Indeed, the substrate cleavage product is expected to peak with a delay relative to the enzyme activity peak. Regarding differences in subcellular distribution of CC3 and the tracer, difference in diffusion speed between a small tracer molecule and a large protein molecule, and caspase translocation between compartments during the progress of apoptosis must be considered. It is also possible that at this stage, the tracer had to compete with other abundant CC3 substrates, such as procaspases.

While Ga-TC3-OGDOTA/[^68^Ga]Ga-TC3-OGDOTA clearly accumulated in apoptotic cells in primary neuronal cultures and in brain tissues of two mouse models of increased apoptosis, we observed some additional, possibly nonspecific, fluorescent objects in both cases. Some of these objects originated from brain tissue autofluorescence and were detected in both conventional 20-µm brain sections and CLARITY-treated 2-mm brain sections (Figure 5, Supplementary Figure 2). These autofluorescent objects may result in false positive results, when no additional immunostaining is performed. However, most often they have non-cell-like morphology (blood vessels, diffused fluorescence) and, therefore, can easily be distinguished from [^68^Ga]Ga-TC3-OGDOTA-positive cells. In cell cultures, Ga-TC3-OGDOTA bound to dead cell debris (Figure 1), which can be distinguished from apoptotic cells by propidium iodide staining, which could be explained by caspase-3 translocation to the nucleus [51]. In 5xFAD brains, [^68^Ga]Ga-TC3-OGDOTA accumulated in well-defined cells, but also abundantly surrounded amyloid plaques and may help in their visualization. These events may suggest some nonspecific interaction between [^68^Ga]Ga-TC3-OGDOTA and native molecules, which could be aided by ionic interactions between the positively charged cell-penetrating peptide of the tracer and the net negative charge of A*β* or DNA. Such interactions have been shown for other proteins with similar peptide sequences, such as the cellular prion protein. Its N-terminal domain contains motifs similar to those of the CPP and binds A*β* with nanomolar affinity [27] as well as DNA and RNA [52, 53]. Due to their lower specificity and/or spatial/morphological features, the identity of such interactions should be readily distinguishable from the desired signal. However, further studies are needed to confirm or reject such nonspecific interactions of Ga-TC3-OGDOTA.

The use of a PET tracer has great potential for translational studies of brain pathologies, but it also brings a number of experimental difficulties. ^68^Ga has a half-life of 68 min, which requires rapid use of the tracer after its generation. The resolution of [^68^Ga]Ga-TC3-OGDOTA distribution in mouse tissues is on the millimeter range, making it difficult to isolate uptake from specific mouse brain structures. The resolution limitation prevented the analysis of uptake from regions of interest such as the ischemic core in the MCAO model and its peripheral areas. Here, we used a simplified assignment of ROIs that allowed us to distinguish injured and uninjured halves of the brains using a minimal number of animals. Nevertheless, standardized uptake varied between mice within each condition, which prohibited distinguishing injured and control brains by absolute uptake values. This difficulty could be related to the fact that the core of the injury might take up even less [^68^Ga]Ga-TC3-OGDOTA than normal tissue due to tissue lysis and may be different between operated animals. Further work to improve the separation of the ischemia core, penumbra, and uninjured tissue regions would simplify the analysis of PET kinetics.

While the bifunctionality of [^68^Ga]Ga-TC3-OGDOTA allows imaging of subcellular compartments to organs and tissues, its radioactivity limited our ability to proceed to fluorescence microscopy immediately after the PET procedure. Indeed, the animals had to be processed in a nonradioactive facility 20 h after the tracer injection, which might have influenced its distribution throughout the brain tissue, particularly, in a fast-developing insult such as stroke. Of note, in this proof-of-principle study, we did not attempt codetection of [^68^Ga]Ga-TC3-OGDOTA by PET and microscopy in the same brain cells, but rather radiolocalization of the tracer in the apoptosis-affected regions of the brain and its further fluorescence localization within CC3-positive brain cells. However, in the future studies, it will be important to shorten the delay between the PET procedure and extraction of the brain for confocal microscopy.

The current study is mainly exploratory, due to the novelty of the tracer and unknown amount of apoptotic neurodegeneration in the disease mouse models used in this work. Therefore, we did not estimate the sample size needed prior to starting these experiments, but rather, based on the obtained results, predicted the necessary sample size for future studies to achieve power of 0.8. For Patlak analysis, we obtained a sample size *N* = 4 for MCAO mice and *N* = 6 for all groups of 5xFAD mice, which is slightly lower than the number of animals used in the current study, except for 5xFAD males. To detect differences between linear decay constants in 5xFAD and wild-type mice, *N* = 3 is needed for all groups. The only comparison that did not include a large enough sample based on postexperiment estimates was the comparison of MCAO ipsilateral and contralateral sides of the brain. This comparison was estimated to need *N* = 16 for the linear decay approximation analyses and *N* = 18 for Patlak analyses. Future improvements in ROI assignment should help reduce these numbers.

Another limitation of this study is the unknown stability of [^68^Ga]Ga-TC3-OGDOTA during the imaging procedure. Future studies should evaluate the stability of the radiotracer under various pH conditions and in blood plasma. It has also been shown that under certain neuropathological conditions, such as AD or stroke, the neurovasculature is affected. Currently, it is unknown whether neurovasculature changes could affect the blood-brain barrier penetration of our tracer. Future studies should directly compare the efficiency of blood-brain barrier crossing of [^68^Ga]Ga-TC3-OGDOTA under different conditions. An arterial input function was not used in the current study. It is possible to approximate the arterial input function given some assumptions, or to measure it directly. However, the former approach has no consensus, and the latter approach is challenging for very small animals (e.g., mice). Finally, a fundamental limitation of ^68^Ga is the high positron range, which effectively reduces resolution compared to ^18^F. Although micro-PET was used in the current study, the use of such a system does not mitigate this specific limitation. The use of micro-PET/CT in the future would improve registration of PET images with images of brain anatomy to study pharmacokinetics in small regions.

In addition to stroke and AD, there are other neurodegenerative conditions featuring significant neuronal loss, including Parkinson and prion diseases. Further work employing [^68^Ga]Ga-TC3-OGDOTA in these disease models is warranted. While the limited number of mice at specific ages in this work did not allow for the analysis of sex-based differences, such comparisons are warranted in future studies to determine whether there are sex-based differences in apoptosis in this AD mouse model. Another application for [^68^Ga]Ga-TC3-OGDOTA could be in the in vivo assessment of therapeutic efficacy in cancer. Considering possible negative side effects on subjects, Ga-TC3-OGDOTA/[^68^Ga]Ga-TC3-OGDOTA did not exert any overt toxicity in neuronal cultures, nor did it cause observable discomfort/disease in the injected mice. Nevertheless, a detailed study of its half-life in mice and possible toxic effects in a second species should be performed prior to clinical studies.

## 5. Conclusions

In conclusion, the caspase-3-targeted [^68^Ga]Ga-TC3-OGDOTA designed in the current study accumulates in neurons expressing increased levels of CC3 indicative of apoptosis. Ga-TC3-OGDOTA/[^68^Ga]Ga-TC3-OGDOTA accumulation was observed in vitro in primary neurons using its fluorescent tag following CPT, OGD, and A*β*O toxicity. Furthermore, it was detected in vivo and ex vivo in the brain in mouse models of cerebral ischemia and Alzheimer's disease, using ^68^Ga-PET and CLARITY imaging. This tracer molecule has many potential applications associated with the early detection of neurodegenerative conditions including Alzheimer's disease, and in the development of novel therapeutics for cancer.

## Figures and Tables

**Table 1:** Summary of the animal use.

	Condition/genotype	Sex	Mice injected with [^68^Ga]Ga-TC3-OGDOTA	Subset used				Mice used for TTC staining	Total number of mice
				PET	Thin sections	CLARITY	Unused		
Stroke model	MCAO	m	8	5	4	3	1^*∗*^	2	10
	Sham-operated controls	m	6	3	3	3	0	—	6

AD model	5xFAD	m	3	3	0	3	0	—	3
		f	7	7	0	3	0	—	7
	WT controls	m	3	3	0	1	0	—	3
		f	6	6	0	1	0	—	6

^*∗*^Died during anaesthesia.

## Data Availability

The fluorescence images and 3D reconstructed videos supporting the findings of this study are included within the supplementary information files. The raw PET and micrograph data used to support the findings of this study are available from the corresponding author upon request.

## Supplementary Materials

Supplementary 1 Supplementary Figure 1. Principle of [^68^Ga]Ga-TC3-OGDOTA retention in apoptotic cells and HPLC verification of its purity. (a) Scheme of [^68^Ga]Ga-TC3-OGDOTA (top) interaction with healthy (bottom left) and apoptotic, containing active/cleaved caspase-3 (CC3, bottom right) cells. (b) HPLC for free Oregon Green conjugate (top), radiolabeled probe 3 (centre), and probe 3 labeled with “cold” nonradioactive Ga^3+^ (bottom) was done as described in Methods (Method C).

Supplementary 2 Supplementary Figure 2. Apoptotic markers in primary neuronal cultures treated with toxic agents. (a, b) Neuronal cultures were treated with 10 *µ*M CPT as described in Methods, and immunostained for cleaved caspase 3 (red) and nuclear marker (DAPI, cyan). Cultures were imaged using 20x (a) or 63x (b) objective on a confocal microscope. (c) Neuronal cultures were treated with OGD for 1 h as described in Methods and immunostained for cleaved caspase 3 (red) and nuclear marker (Hoechst, blue). Cultures were imaged using 20x objective on a FV1000 Olympus confocal microscope. (d) Neuronal cultures were treated with A*β*Os for 24 h as described in Methods and immunostained for cleaved caspase 3 (red) and nuclear marker (Hoechst, blue). Cultures were imaged using 60x objective on a FV1000 Olympus confocal microscope.

Supplementary 3 Supplementary Figure 3. Confocal and CLARITY imaging of MCAO mouse brains. (a) TTC staining of MCAO mouse brains reveals variations in the ischemic injury core size. (b) Sample kinetics for PET imaging of [^68^Ga]Ga-TC3-OGDOTA injected in a 3-month-old male mouse. SUV was calculated for brain, bladder, kidney, liver, hip, and left ventricle as PET signal integrated over the region of interest and normalized by ID/weight (g). SUV is presented as mean ± SD. (c) Comparison of Oregon Green imaging (ex505/em525-560) in MCAO mouse brains injected (left panel) or not (two right panels) with [^68^Ga]Ga-TC3-OGDOTA. White arrows point at [^68^Ga]Ga-TC3-OGDOTA-positive cells, wide green arrow—at nonspecific green glare in MCAO tissue, thin green arrows—at self-fluorescing blood vessels. (d) Neuronal cultures treated with OGD and incubated for 7 h under normal conditions with 1 *µ*M [^68^Ga]Ga-TC3-OGDOTA were extensively washed for 72 h with clearing solution (4% SDS in 200 mM sodium borate, pH 8.5) at 37 °C or stored at 37 °C without washing (control). Imaging was performed using an FV1000 confocal microscope equipped with 30x objective. (e) CLARITY steps: left—2 mm coronal brain sections after 72 h clearing as described in Methods; right—the same section after incubation for 2 h in SeeDB mounting medium and mounting on a slide.

Supplementary 4 Supplementary video 1. 3D reconstruction of Z-stack confocal images of a 0.7 mm thick coronal section (cortical area) of MCAO-operated mouse after [^68^Ga]Ga-TC3-OGDOTA (Oregon Green = green) injection. Operated side is on the left in the beginning of the video.

Supplementary 5 Supplementary video 2. 3D reconstruction of Z-stack confocal images of a 0.7 mm thick coronal section (midbrain area) of MCAO-operated mouse after [^68^Ga]Ga-TC3-OGDOTA (Oregon Green = green) injection. Operated side is on the left in the beginning of the video.

Supplementary 6 Supplementary video 3. 3D reconstruction of Z-stack confocal images of a 0.7 mm thick coronal section (cortical area) of MCAO-operated mouse after [^68^Ga]Ga-TC3-OGDOTA injection and immunostaining for CC3 (gold). Operated side is on the left.

Supplementary 7 Supplementary video 4. 3D reconstruction of Z-stack confocal images of a 0.7 mm thick coronal section (midbrain area) of MCAO-operated mouse after [^68^Ga]Ga-TC3-OGDOTA injection and immunostaining for CC3 (gold). Operated side is on the left in the beginning of the video.

Supplementary 8 Supplementary video 5. 3D reconstruction of Z-stack confocal images of 0.2 mm thick coronal sections of 5xFAD mouse brains (bottom) and its wild-type control (top) after [^68^Ga]Ga-TC3-OGDOTA (Oregon Green = green) injection.

Supplementary 9 Supplementary video 6. 3D reconstruction of Z-stack confocal images of a 0.2 mm-thick coronal section (hippocampus area) of 5xFAD mouse brains after [^68^Ga]Ga-TC3-OGDOTA (Oregon Green=green) injection.

Supplementary 10 Supplementary video 7. 3D reconstruction of Z-stack confocal images of a half of a 0.2 mm thick coronal section of 5xFAD mouse brains after [^68^Ga]Ga-TC3-OGDOTA (Oregon Green = yellow) injection and staining with Thioflavin S (blue) for amyloid plaques. The microscope was equipped with a 10x objective lens.

Supplementary 11 Supplementary video 8. 3D reconstruction of Z-stack confocal images of a 0.4 mm-thick coronal section (hippocampal area) of 5xFAD mouse brains after [^68^Ga]Ga-TC3-OGDOTA (Oregon Green = yellow) injection and staining with Thioflavin S (blue) for amyloid plaques. The microscope was equipped with a 30x objective lens.

## References

[1] Barthel H., Schroeter M. L., Hoffmann K.-T., Sabri O. (2015). PET/MR in dementia and other neurodegenerative diseases. *Seminars in nuclear medicine*.

[2] Del Sole A., Malaspina S., Magenta Biasina A. (2016). Magnetic resonance imaging and positron emission tomography in the diagnosis of neurodegenerative dementias. *Functional neurology*.

[3] Wey H.-Y., Desai V. R., Duong T. Q. (2013). A review of current imaging methods used in stroke research. *Neurological research*.

[4] Hoglund J., Shirvan A., Antoni G. (2011). 18F-ML-10, a PET tracer for apoptosis: first human study. *Journal of Nuclear Medicine*.

[5] Elmore S. (2016). Apoptosis: a review of programmed cell death. *Toxicologic pathology*.

[6] Sebbagh M., Renvoizé C., Hamelin J., Riché N., Bertoglio J., Bréard J. (2001). Caspase-3-mediated cleavage of ROCK I induces MLC phosphorylation and apoptotic membrane blebbing. *Nature cell biology*.

[7] Wolf B. B., Schuler M., Echeverri F., Green D. R. (1999). Caspase-3 is the primary activator of apoptotic DNA fragmentation via DNA fragmentation factor-45/inhibitor of caspase-activated DNase inactivation. *Journal of Biological Chemistry*.

[8] Challapalli A., Kenny L. M., Hallett W. A. (2013). 18F-ICMT-11, a caspase-3-specific PET tracer for apoptosis: biodistribution and radiation dosimetry. *Journal of Nuclear Medicine*.

[9] Limpachayaporn P., Wagner S., Kopka K., Hermann S., Schäfers M., Haufe G. (2013). Synthesis, 18F-radiolabeling, and in vivo biodistribution studies of N-fluorohydroxybutyl isatin sulfonamides using positron emission tomography. *Journal of medicinal chemistry*.

[10] Hickson J., Ackler S., Klaubert D. (2010). Noninvasive molecular imaging of apoptosis in vivo using a modified firefly luciferase substrate, Z-DEVD-aminoluciferin. *Cell Death and Differentiation*.

[11] Zhang L., Xu Q., Xing D., Gao C., Xiong H. (2009). Real-time detection of caspase-3-like protease activation in vivo using fluorescence resonance energy transfer during plant programmed cell death induced by ultraviolet C overexposure. *Plant physiology*.

[12] Nicholls P., Pack T., Urs N (2017). Mapping nonapoptotic caspase activity with a transgenic reporter in mice. *bioRxiv*.

[13] Bullok K. E., Maxwell D., Kesarwala A. H. (2007). Biochemical andin VivoCharacterization of a small, membrane-permeant, caspase-activatable far-red fluorescent peptide for imaging apoptosis†. *Biochemistry*.

[14] Kim K., Lee M., Park H. (2006). Cell-permeable and biocompatible polymeric nanoparticles for apoptosis imaging. *Journal of the American Chemical Society*.

[15] Maxwell D., Chang Q., Zhang X., Barnett E. M., Piwnica-Worms D. (2009). An improved cell-penetrating, caspase-activatable, near-infrared fluorescent peptide for apoptosis imaging. *Bioconjugate Chemistry*.

[16] Ye D., Shuhendler A. J., Cui L. (2014). Bioorthogonal cyclization-mediated in situ self-assembly of small-molecule probes for imaging caspase activity in vivo. *Nature Chemistry*.

[17] Engel B. J., Gammon S. T., Chaudhari R. (2018). Caspase-3 substrates for noninvasive pharmacodynamic imaging of apoptosis by PET/CT. *Bioconjugate Chemistry*.

[18] Rapic S., Vangestel C., Elvas F. (2017). Evaluation of [18F]CP18 as a substrate-based apoptosis imaging agent for the assessment of early treatment response in oncology. *Molecular Imaging and Biology*.

[19] Su H., Chen G., Gangadharmath U. (2013). Evaluation of [18F]-CP18 as a PET imaging tracer for apoptosis. *Molecular Imaging and Biology*.

[20] Palner M., Shen B., Jeon J., Lin J., Chin F. T., Rao J. (2015). Preclinical kinetic analysis of the caspase-3/7 PET tracer 18F-C-SNAT: quantifying the changes in blood flow and tumor retention after chemotherapy. *Journal of Nuclear Medicine*.

[21] Snir J. A., Suchy M., Lawrence K. S., Hudson R. H. E., Pasternak S. H., Bartha R. (2015). Prolonged in vivo retention of a cathepsin D targeted optical contrast agent in a mouse model of Alzheimer’s disease. *Journal of Alzheimer’s Disease*.

[22] Snir J. A., Suchy M., Bindseil G. A. (2018). An aspartyl cathepsin targeted PET agent: application in an Alzheimer’s disease mouse model. *Journal of Alzheimer’s Disease*.

[23] Ta R., Suchy M., Tam J. H. K. (2012). A dual magnetic resonance imaging/fluorescent contrast agent for Cathepsin-D detection. *Contrast Media and Molecular Imaging*.

[24] Suchý M., Ta R., Li A. X. (2010). A paramagnetic chemical exchange-based MRI probe metabolized by cathepsin D: design, synthesis and cellular uptake studies. *Organic and Biomolecular Chemistry*.

[25] Stennicke H. R., Renatus M., Meldal M., Salvesen G. S. (2000). Internally quenched fluorescent peptide substrates disclose the subsite preferences of human caspases 1, 3, 6, 7 and 8. *Biochemical Journal*.

[26] Brom M., Franssen G. M., Joosten L., Gotthardt M., Boerman O. C. (2016). The effect of purification of Ga-68-labeled exendin on in vivo distribution. *EJNMMI Research*.

[27] Ostapchenko V. G., Beraldo F. H., Mohammad A. H. (2013). The prion protein ligand, stress-inducible phosphoprotein 1, regulates amyloid- oligomer toxicity. *Journal of Neuroscience*.

[28] Beraldo F. H., Soares I. N., Goncalves D. F. (2013). Stress-inducible phosphoprotein 1 has unique cochaperone activity during development and regulates cellular response to ischemia via the prion protein. *The FASEB Journal*.

[29] Um J. W., Nygaard H. B., Heiss J. K. (2012). Alzheimer amyloid-*β* oligomer bound to postsynaptic prion protein activates Fyn to impair neurons. *Nature Neuroscience*.

[30] Larson M., Sherman M. A., Amar F. (2012). The complex PrPc-Fyn couples human oligomeric a with pathological tau changes in Alzheimer’s disease. *Journal of Neuroscience*.

[31] Longa E. Z., Weinstein P. R., Carlson S., Cummins R. (1989). Reversible middle cerebral artery occlusion without craniectomy in rats. *Stroke*.

[32] Zarow G., Karibe H., States B., Graham S., Weinstein P. (2016). Endovascular suture occlusion of the middle cerebral artery in rats: effect of suture insertion distance on cerebral blood flow, infarct distribution and infarct volume. *Neurological Research*.

[33] Broughton B. R. S., Reutens D. C., Sobey C. G. (2009). Apoptotic mechanisms after cerebral ischemia. *Stroke*.

[34] Loo D. T., Copani A., Pike C. J., Whittemore E. R., Walencewicz A. J., Cotman C. W. (1993). Apoptosis is induced by beta-amyloid in cultured central nervous system neurons. *Proceedings of the National Academy of Sciences*.

[35] Selznick L. A., Holtzman D. M., Han B. H. (1999). In situ immunodetection of neuronal caspase-3 activation in Alzheimer disease. *Journal of Neuropathology and Experimental Neurology*.

[36] Bilkei-Gorzo A. (2014). Genetic mouse models of brain ageing and Alzheimer’s disease. *Pharmacology and Therapeutics*.

[37] Patlak C. S., Blasberg R. G., Fenstermacher J. D. (2016). Graphical evaluation of blood-to-brain transfer constants from multiple-time uptake data. *Journal of Cerebral Blood Flow and Metabolism*.

[38] Peters A. M. (1994). Graphical analysis of dynamic data. *Nuclear Medicine Communications*.

[39] Oakley H., Cole S. L., Logan S. (2006). Intraneuronal beta-amyloid aggregates, neurodegeneration, and neuron loss in transgenic mice with five familial Alzheimer’s disease mutations: potential factors in amyloid plaque formation. *Journal of Neuroscience*.

[40] Rojas S., Herance J. R., Gispert J. D. (2013). In vivo evaluation of amyloid deposition and brain glucose metabolism of 5XFAD mice using positron emission tomography. *Neurobiology of Aging*.

[41] Ostapchenko V. G., Beraldo F. H., Guimarães A. L. S. (2013). Increased prion protein processing and expression of metabotropic glutamate receptor 1 in a mouse model of Alzheimer’s disease. *Journal of Neurochemistry*.

[42] Ostapchenko V. G., Chen M., Guzman M. S. (2015). The transient receptor potential melastatin 2 (TRPM2) channel contributes to -amyloid oligomer-related neurotoxicity and memory impairment. *Journal of Neuroscience*.

[43] Chung K., Deisseroth K. (2013). CLARITY for mapping the nervous system. *Nature Methods*.

[44] Ke M.-T., Fujimoto S., Imai T. (2013). SeeDB: a simple and morphology-preserving optical clearing agent for neuronal circuit reconstruction. *Nature Neuroscience*.

[45] Morris E. J., Geller H. M. (1996). Induction of neuronal apoptosis by camptothecin, an inhibitor of DNA topoisomerase-I: evidence for cell cycle-independent toxicity. *The Journal of Cell Biology*.

[46] Ishikawa H., Tajiri N., Vasconcellos J. (2013). Ischemic stroke brain sends indirect cell death signals to the heart. *Stroke*.

[47] Popp A., Jaenisch N., Witte O. W., Frahm C. (2009). Identification of ischemic regions in a rat model of stroke. *PloS One*.

[48] Malagelada C., Xifro X., Minano A., Sabria J., Rodriguezalvarez J. (2005). Contribution of caspase-mediated apoptosis to the cell death caused by oxygen-glucose deprivation in cortical cell cultures. *Neurobiology of Disease*.

[49] Kadowaki H., Nishitoh H., Urano F. (2005). Amyloid *β* induces neuronal cell death through ROS-mediated ASK1 activation. *Cell Death and Differentiation*.

[50] Yu M.-S., Suen K.-C., Kwok N.-S., So K.-F., Hugon J., Chuen-Chung Chang R. (2006). Beta-amyloid peptides induces neuronal apoptosis via a mechanism independent of unfolded protein responses. *Apoptosis*.

[51] Kamada S., Kikkawa U., Tsujimoto Y., Hunter T. (2004). Nuclear translocation of caspase-3 is dependent on its proteolytic activation and recognition of a substrate-like protein(s). *Journal of Biological Chemistry*.

[52] Gabus C., Auxilien S., Péchoux C. (2001). The prion protein has DNA strand transfer properties similar to retroviral nucleocapsid protein 1 1Edited by J. Karn. *Journal of molecular biology*.

[53] Yin S., Fan X., Yu S., Li C., Sy M.-S. (2008). Binding of recombinant but not endogenous prion protein to DNA causes DNA internalization and expression in mammalian cells. *Journal of Biological Chemistry*.

