# Supplementary figures and images for: Detection of Active Caspase-3 in Mouse Models of Stroke and Alzheimer's Disease with a Novel Dual Positron Emission Tomography/Fluorescent Tracer [68Ga]Ga-TC3-OGDOTA
Source: Contrast Media Mol Imaging. 2019 Jan 14;2019:6403274. doi: 10.1155/2019/6403274 (PMC6348924; doi:10.1155/2019/6403274)

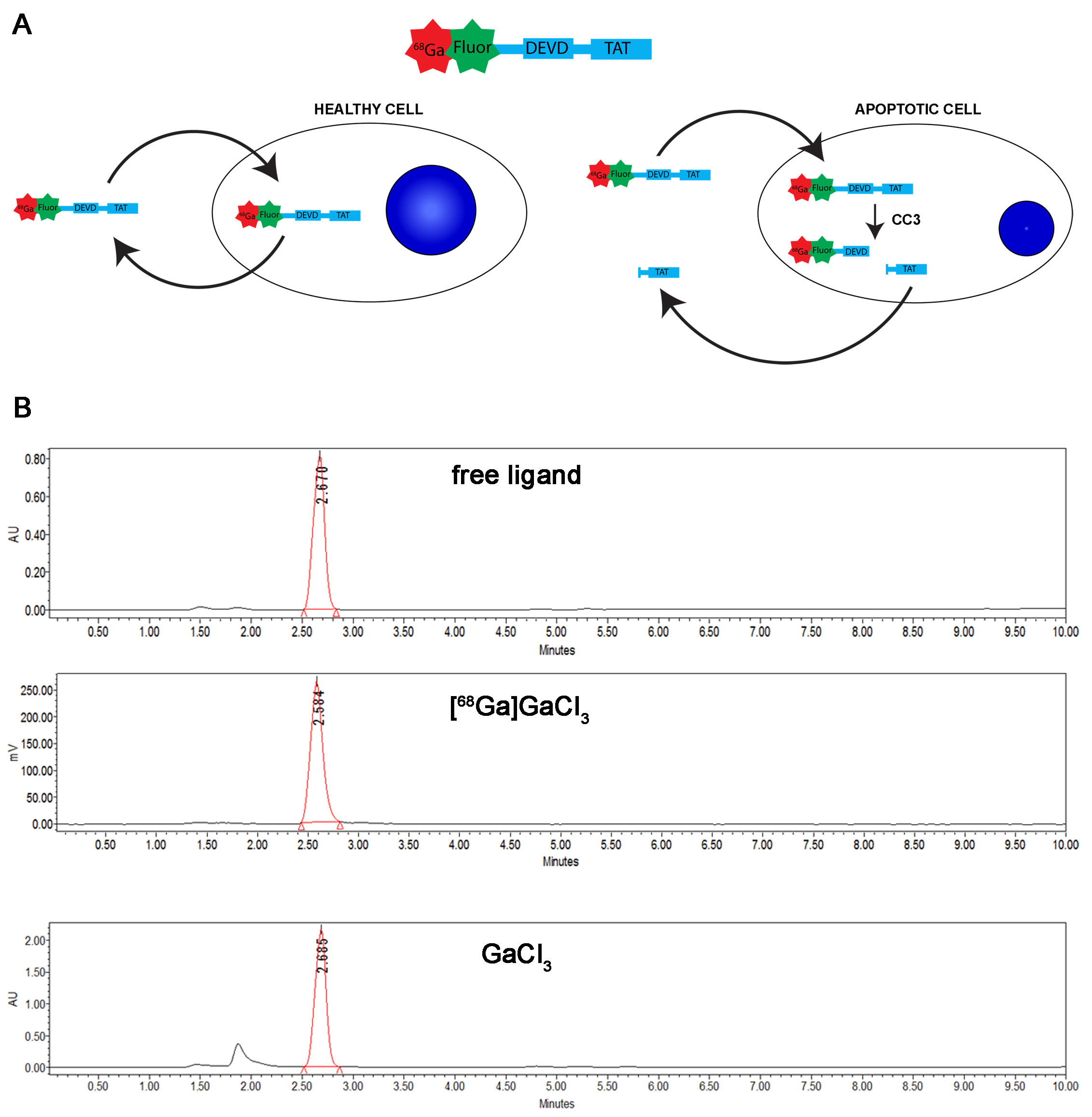

Supplement: Supplementary 1 — Supplementary Figure 1. Principle of [68Ga]Ga-TC3-OGDOTA retention in apoptotic cells and HPLC verification of its purity. (a) Scheme of [68Ga]Ga-TC3-OGDOTA (top) interaction with healthy (bottom left) and apoptotic, containing active/cleaved caspase-3 (CC3, bottom right) cells. (b) HPLC for free Oregon Green conjugate (top), radiolabeled probe 3 (centre), and probe 3 labeled with “cold” nonradioactive Ga3+ (bottom) was done as described in Methods (Method C). [file 6403274.f1.jpg]

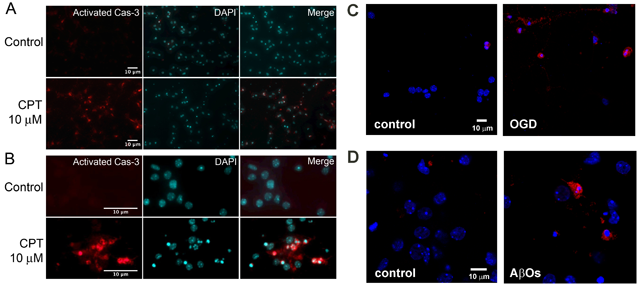

Supplement: Supplementary 2 — Supplementary Figure 2. Apoptotic markers in primary neuronal cultures treated with toxic agents. (a, b) Neuronal cultures were treated with 10 µM CPT as described in Methods, and immunostained for cleaved caspase 3 (red) and nuclear marker (DAPI, cyan). Cultures were imaged using 20x (a) or 63x (b) objective on a confocal microscope. (c) Neuronal cultures were treated with OGD for 1 h as described in Methods and immunostained for cleaved caspase 3 (red) and nuclear marker (Hoechst, blue). Cultures were imaged using 20x objective on a FV1000 Olympus confocal microscope. (d) Neuronal cultures were treated with AβOs for 24 h as described in Methods and immunostained for cleaved caspase 3 (red) and nuclear marker (Hoechst, blue). Cultures were imaged using 60x objective on a FV1000 Olympus confocal microscope. [file 6403274.f2.tif]

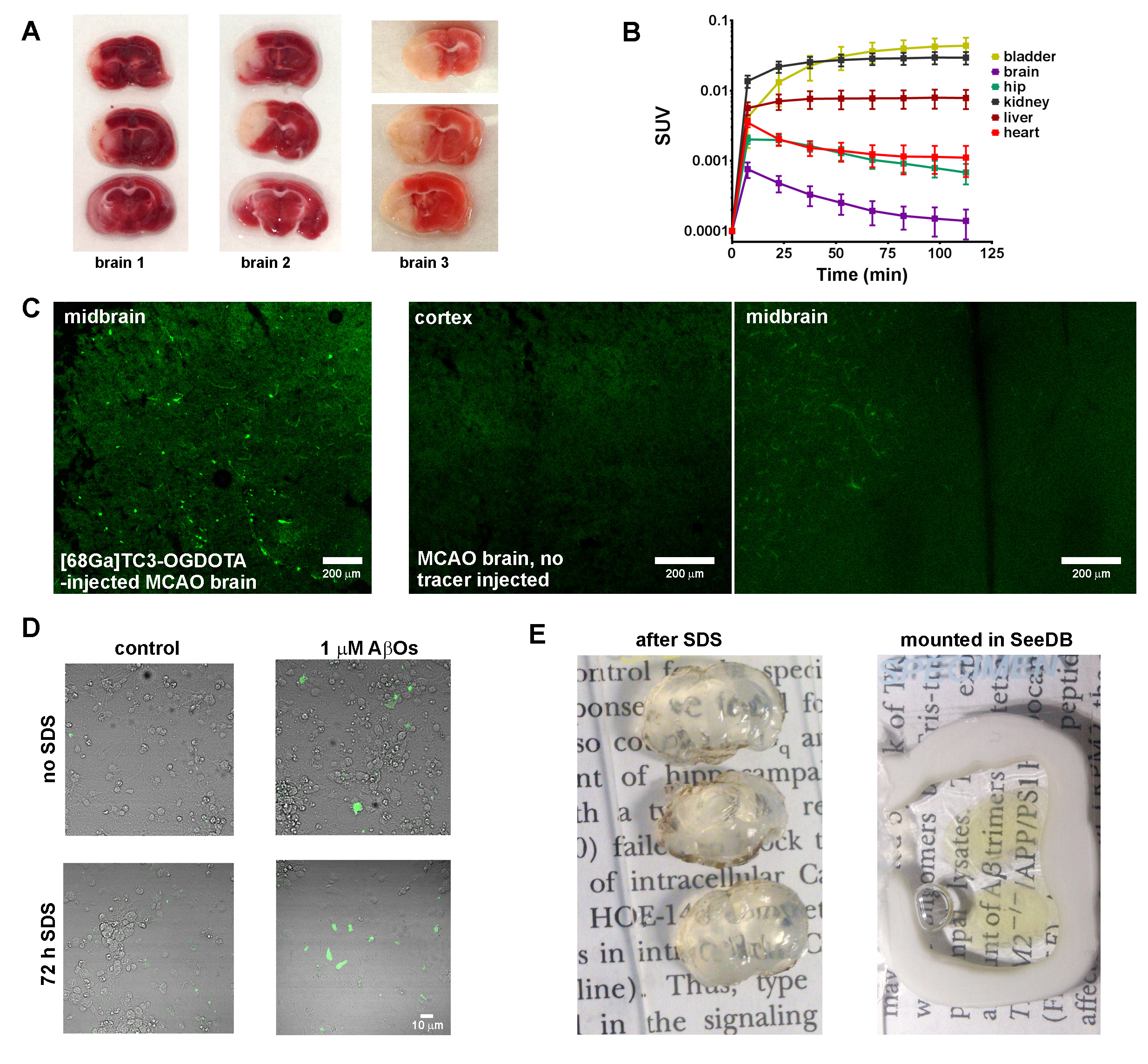

Supplement: Supplementary 3 — Supplementary Figure 3. Confocal and CLARITY imaging of MCAO mouse brains. (a) TTC staining of MCAO mouse brains reveals variations in the ischemic injury core size. (b) Sample kinetics for PET imaging of [68Ga]Ga-TC3-OGDOTA injected in a 3-month-old male mouse. SUV was calculated for brain, bladder, kidney, liver, hip, and left ventricle as PET signal integrated over the region of interest and normalized by ID/weight (g). SUV is presented as mean ± SD. (c) Comparison of Oregon Green imaging (ex505/em525-560) in MCAO mouse brains injected (left panel) or not (two right panels) with [68Ga]Ga-TC3-OGDOTA. White arrows point at [68Ga]Ga-TC3-OGDOTA-positive cells, wide green arrow—at nonspecific green glare in MCAO tissue, thin green arrows—at self-fluorescing blood vessels. (d) Neuronal cultures treated with OGD and incubated for 7 h under normal conditions with 1 µM [68Ga]Ga-TC3-OGDOTA were extensively washed for 72 h with clearing solution (4% SDS in 200 mM sodium borate, pH 8.5) at 37 °C or stored at 37 °C without washing (control). Imaging was performed using an FV1000 confocal microscope equipped with 30x objective. (e) CLARITY steps: left—2 mm coronal brain sections after 72 h clearing as described in Methods; right—the same section after incubation for 2 h in SeeDB mounting medium and mounting on a slide. [file 6403274.f3.jpg]
